# Annotations

**Published:** 1893-04-01

**Authors:** 


### Annotations.

Sir Andrew Clark is to be congratulated upon his election, for the sixth year in succession, to the presidency of the Royal College of Physicians; and the College is also to be congratulated in that it has shown a sure instinct for the man and the moment. It is an old truth that "no man liveth to himself"; it is a somewhat modern experience that no college or public institution can any longer "live to itself." The days of close corporations are unalterably numbered. The College has always filled a considerable place in the estimation of the medical profession. It is now called upon to fill, not a considerable but a great place; and not in the estimation alone of medical practitioners, who are a mere handful, but of the whole cultured population of this vast empire. Physic has at last "arrived," to use an expressive Americanism. She is one of the conspicuous forces and leaders of science, and of the culture which is peculiar to science. She, therefore, demands to be represented by men of the modern knowledge, the modern culture, and the modern spirit. Such a man is Sir Andrew Clark. Happily for Sir Andrew he has his enemies, and to them we will leave the task of insisting upon the obverse of the shield. We have only space for a brief congratulation, not for a critical estimate. Even, however, if we were called upon for a critical estimate we see no reason for any other general conclusions than those already indicated. Sir Andrew Clark is excellently fitted to represent medicine to medicine, and also to represent it to the world. By diligence and duty he won his first honours at the College; by diligence and duty he has retained the suffrages of its fellows. There is no other way to honour so entirely honourable as this.

**The Presidency of the R. C. P.**

Dr. Klein has laid the medical profession and the public under obligations by the careful thoroughness with which he has repeated Haffkine's cholera inoculations, and by the additional light which he has thrown upon the whole subject. In the first place it is manifest that the so-called cholera bacillus has not as yet quite made out its claim to be the one and only cause of the disease known as Asiatic cholera. Dr. Klein, experimenting with this, and with five other distinct varieties of bacillus, produced in the animals experimented upon a series of symptoms which are quite identical in each case. The six varieties of bacillus employed were: (1) The cholera bacillus, (2) Finkler's vibrio, (3) bacillus coli, (4) bacillus prodigiosus, (5) proteus vulgaris, and (6) the typhoid bacillus. The animals experimented upon were guinea pigs; and the experiments consisted of intra-peritoneal injections of agar-cultures of the six varieties of bacillus. The results of the injections were that as a rule every unprotected animal injected was found dead in from twenty to twenty-four hours. The appearances after death, in all the cases, were intense peritonitis, membranous exudations on the liver and omentum, congestion of intestine and serous surfaces, the peritoneum contained turbid, sanguineous, fluid exudation filled with microbes, and the blood of the heart, and of course therefore of other parts, also contained microbes. The interesting point is that small protective injections of any of these bacilli rendered animals immune against any or all of the others, and what is more, even against Haffkine's "Virus forte." What, then, is the practical conclusion? That Haffkine's cholera injections are absolutely protective, and that injections of the other four or five bacilli named are also protective? By no means! For the bacilli themselves constitute one kind of poison, and the ptomaines they elaborate constitute another and very different kind. An animal, it appears, may after inoculation be perfectly immune against actual bacilli, of cholera or other disease-producing varieties, but may be as susceptible to the ptomaines or other toxins produced and elaborated by the bacilli as if it had never been inoculated at all. Inoculated animals, which successfully resisted Haffkine's "Virus forte," died promptly when afterwards inoculated with gelatine culture (liquified by growth) of the so-named cholera bacillus. Are we, then, where we were? By no means! Knowledge is steadily growing, and facts are being systematically differentiated. Our next step must be to find antidotes for the ptomaines and other toxins which the bacilli elaborate in the culture media, and of course as they develop in the animal body. When that is done we shall be a step nearer our ultimate goal of protective inoculation.

**Dr. Klein and Cholera Inoculations.**

Apropos of a recent legal decision, a lady correspondent writes: "Knowing the waste of mind, body, and estate entailed upon myself through reading ——s delusive pamplet advising educated women wishing for useful work to train in electro-massage, I have long and earnestly protested against the whole subject, and it only remains for massage to be shown up." Our correspondent goes on to explain that she believes in electro-therapeutics and also in massage, either separately or in combination. What she does not believe in—what on the contrary she detests and regards with horror, is the way in which these systems of medical treatment are often employed by designing and wicked persons—in the fleecing of patients and the destruction of their self-respect. This same correspondent has several times during the past three years addressed us on what she evidently considers a burning question. We have every reason to believe that she speaks from an intimate knowledge of the subject and of persons engaged in these methods of treatment. But what is it of a practical nature that we can do? Medical men of worthy character burn with shame when they think of certain things which are done under the name of therapeutics. But their shame leads to no result whatever. The people who ought to be ashamed laugh at the very idea of shame. The medical profession is very sharply divided into two classes: those who practice their art with honest and intelligent simplicity, and those who, on the other hand, cynically subordinate their calling to the making of the largest possible amount of money out of it—honestly or otherwise. If our correspondent, or any other person, cares to furnish us with definite names, dates, and facts, we shall not hesitate to publish them and to do what is in our power to bring unscrupulous masseurs to book. Moreover, we shall, in any case, take such steps as may be possible to discover what amount of truth there may be in these repeated charges. There is no kind of terrorism which causes more suffering and depravation of character than the terrorism exercised by unscrupulous practitioners of the medical art over their deluded and most unhappy victims.

**Is Massage a Swindle?**

